# Sustainability-Sport-Physical Activity

**DOI:** 10.3390/ijerph18041455

**Published:** 2021-02-04

**Authors:** Éva Bácsné-Bába, Gergely Ráthonyi, Christa Pfau, Anetta Müller, György Norbert Szabados, Mónika Harangi-Rákos

**Affiliations:** 1 Institute of Sport Management, University of Debrecen, H-4032 Debrecen, Hungary; bacsne.baba.eva@econ.unideb.hu (É.B.-B.); pfau.christa@econ.unideb.hu (C.P.); muller.anetta@econ.unideb.hu (A.M.); 2 Institute of Applied Informatics and Logistics, University of Debrecen, H-4032 Debrecen, Hungary; 3 Institute of Management and Organization Sciences, University of Debrecen, H-4032 Debrecen, Hungary; szabados.gyorgy@econ.unideb.hu; 4 Institute of Rural Development, Regional Economy and Tourism Management, University of Debrecen, H-4032 Debrecen, Hungary; rakos.monika@econ.unideb.hu

**Keywords:** sport, sustainability, living environment, health, activity, lifestyle, awareness

## Abstract

The present study is a synthesizing analysis of international literature on correlations between sustainability, sport, and physical activity. The study of sustainability is considered essential in modern research; its multidisciplinary approach relies on sports science and sports economics as well. There are several aspects of sustainability that are closely associated with health and health preservation; the beneficial effect of exercise on health is also widely known. For the analysis of this complex matter, our current study relied on secondary sources, and besides exploring specialist literature, it also illustrates and analyzes related statistical data. Our results highlight the correlations between living environment and physical activity, the importance of increasing individual commitment towards sustainability and using green spaces for exercising, as well as questions on social and environmental development in relation to physical activity. The study revealed the existence of highly complex links between physical environment, physical activities, and sustainability. The results section of our study provides a brief summary on the possible ways of making people physically active. Increasing physical activity is of paramount importance for achieving the objectives formulated in relation to sustainability.

## 1. Introduction

Nowadays, sustainability and sustainable development are widely used phrases that have become almost trivial. We consider it important, however, to highlight that sustainability means a strive towards development pathways that can be pursued continuously without compromising the reserves and opportunities of future generations and forms of life. Sustainability can be interpreted not only in social, economic, and environmental terms, but also at the international, national, regional, local, and organizational level [1]. Weak sustainability supporters agree that these three pillars are equally important; on the other hand, they also claim that development cannot lead to a decrease in the total amount of economic, social, and environmental capital [2]. In a broader sense, sustainability can be interpreted as the environmental dimension of sustainable development, while in the strict sense, the principle of sustainability is interpretable in terms of processes [3]. In the present article, we accept the common definition of sustainability as a phenomenon that is described with regards to the needs of future generations. People that are currently living on Earth need to pay attention to the needs of future generations while they are covering their own needs in all cases [4]. The current meaning of the notion “sustainability” is a result of continuous improvement, a kind of evolution. The result of this tendency is a notion that is valid for several aspects of the world [5,6,7]. As most people tend to associate the notion of sustainability with environmental aspects [8], the present study also focuses on the associations between sport and environmental sustainability. The relationship between sport and sustainable development is undisputed and quite diverse. Nowadays the connection of the physical activities and the nature is widely recognized and this tendency has a positive effect on the recognition of environmental circumstances as factors influencing the entire sports life concept [9]. Consequently, in the literature of sports sciences, increasing attention is notably being paid to sustainability in sport, and managers involved in competitive sports also focus more and more on sustainability [10]. Due to the nature of the topic, the study of sustainability requires conducting long-term assessments, where the examined sample needs to be re-assessed from different aspects from time to time, and the results need to be further analyzed. This, however, is hindered by the fact that research funds are rarely available for the study of a specific aspect for such a long time that would yield measurable results in activities implemented with the aim of reaching sustainability [11].

When examining the relationship between sustainability and quality of life or sport, we can draw the conclusion that sustainable development is aimed at protecting and preserving our existing natural and cultural values, as well as the economic and technological achievements of our civilization, which ultimately refers to a desire closely associated with our quality of life. To put it simply, our subjective quality of life is reflected in our health, which—as we all know—has a variety of physical, social, and emotional aspects. Quality of life is a complex construct; it involves both the physical, mental, and social wellbeing of society and its individual members. It means the level of adaptation achievement that individuals can obtain when adapting to their physical, biological, social, and economic environment, and their changes. Since the turn of the millennium, research conducted in the field of quality of life, especially the subjective quality of life, has been of paramount importance. The leading experts of this topic consider subjective quality of life to refer to how individuals assess their own lives, whereas others [12,13] regard it as a synonym of happiness. Participation in different physical activities and in sports has a positive effect on the health status of people. Physically active people can obtain several health benefits from it. Physical activities are able to decrease mortality rates as well [14,15,16]. On the other hand, the positive effects of physical activities can be seen in the case of psychological aspects as well. Physical activities support the mental well-being and the social aspects of individuals as well by strengthening their social relations. They also have a positive effect on self-evaluation and on the overall satisfaction with life [17,18,19]. The results of research on the subjective quality of life have also demonstrated that it not only affects the physical or mental health of an individual, but also affects their social, demographic, and economic position, and the quality of their social relationships [20,21,22,23,24,25]. The socioeconomic status plays a crucial role in the case of participation in physical activities. People with a lower socioeconomic status participate in physical activities less often, while people with a higher socioeconomic status usually participate more often in these activities and in sports [26,27]. The reasons for lower participation rates in the case of a lower socioeconomic status are a lack of free time and a lack of available money [28].

Studies on the quality of life are gaining increasing importance in the social sciences. These studies provide both subjective and objective evidence regarding the implementation and efficacy of different policies (economic, education, health, transport, etc.). The cross-European study “Quality of life in Europe” conducted in 2003 assessed and evaluated the health of the European population, the level of their subjective quality of life, and their level of satisfaction regarding services, as well as their time management.

Sustainability criteria play an important part in more and more health and regional development policies; these criteria appear as targets in several special and regional Agenda 21 frameworks. Such frameworks are Health 21, and Local Agenda 21. The Healthy Cities Program (2002) considers the notions of health and sustainable development to be closely related and overlapping constructs. Nowadays, when most of the population lives in urban areas, i.e., in cities, it has become necessary to develop concepts and action plans that support individuals in having a healthier lifestyle and practicing more environmental awareness. These projects aim to make settlements more livable by creating a sustainable infrastructure with consumer and environment-friendly services that promote physical activities, as well as increasing community participation. Hopefully, recognizing the effect of social and economic policies and practices on health will have a long-term impact on decision-making regarding societal development. To enhance these initiatives, a number of educational programs have been launched all over the world to help students learn how sport sciences can promote the advancement of sustainable development [29].

Sport plays an inevitable role in sustainable development and the long-term provision of an ever-improving quality of life. We can analyze physical activities and sports from the viewpoint of social capital as well. The importance of social capital appears on every level of the society from the level of individual persons to the global level. The value of social capital can be developed by the development of the health status of people. In this case, the health status can become one of the fundamentals of the sustainability because health relief means covering the needs of the people actually living on the planet but the prevention of the emergence of health problems in the first place has a positive effect on the future perspectives of the next generations. This role is obvious for example in the case of winter sports or water sports, since the environment needed for them either involves nature itself or some kind of imitation of the natural environment. In other sports, this relationship may be less obvious; fresh air, healthy and sustainable nutrition, and predictable weather are necessary for training; however, they are also essential for the successful pursuit of almost all sports [30]. As an activity, sport also shapes the environment: exercising also requires different devices and apparatus—for example sport equipment, the production and trade of which makes use of environmental resources. In addition, sport itself can affect the environment, for example in the case of sports that can be done in places far away from the participant’s residence (e.g., skiing, surfing); these sports themselves would barely affect the environment [31].

The Organization for Economic Co-operation and Development (OECD) measures what percentage of their daily time people spend doing different activities (a day consists of 1440 min; Table 1 shows the percentage breakdown by activities). Time use was divided into categories like time spent on (paid) work or learning, on unpaid work, time spent on personal care, free time, and other time. The amount of time spent on free time activities—which is the most relevant category for us—was the highest in Norway, Greece, Belgium, and Germany (~26%, ~24%, ~24%, and ~23%, respectively). The next group in line mostly comprised European countries, where the percentage of time spent on free time activities was around 21–22%. This percent was even lower, below 20%, for countries like Turkey, Hungary, Australia, Canada, Japan, and Luxemburg. The lowest share was measured in Mexico (~12%), where the percentage was half that of the countries at the top of the ranking. Similarly, low values were seen in Korea, India, Lithuania, Portugal, and China (15–18%). Surprisingly, there was no clear trend among countries; they showed a heterogeneous distribution instead. The results imply that country specific factors must play a major role here. On average, it was the time spent on personal care that had the highest score—nearly 46%—among the five categories, which was followed by free time activities with a score of 20%. The proportion of paid work or learning was around 19%, unpaid work had 14%, and the percentage of time spent on other activities was a bit above 1%.

## 2. Materials and Methods 

This study examines the relationship between sports or physical activity in general, and sustainability (as defined sustainability related to physical activities). The field is relatively recent; thus, no strict criteria were applied when selecting the studies to be processed. In almost all cases, this subfield was not analyzed in itself, but as a (many times marginally) related form of research. In most cases, the research aim was not to analyze the relationship between sports or physical activity in general and sustainability, which restricted the available and potentially useful studies for this review. We attempted to avoid the subjectivity bias by including all of the relevant studies, even marginally related ones. The sample period was set between 2000 and 2020 in order to cover the relevant studies. However, most of the related studies were only published after 2010. Scientific journal articles were retrieved from the ScienceDirect and Google Scholar databases. We used these sources, since both ScienceDirect and Google Scholar provide immediate access to the relevant studies, while the latter was useful for detecting other types of scientific literature (reports and case studies for example). During the search, the following keywords were used: physical activity, sport activity, sport and sustainability, physical activity and sustainability, sport and environment, physical activity and environment.

All articles whose topic was related in terms of the present study were taken into consideration. Out of the international databases available, we decided to rely more heavily on OECD and EUROSTAT, especially the Eurobarometer Public Opinion Survey. We chose these databases because they provided wide international coverage, while the Eurobarometer provided information about very different subfields as well in terms of physical activity. Results were grouped along several factors in order to provide a clearer overview and a consistent structure. During the completion of our research plan, we followed several listed aims. First, the relationship between sports and sustainability was presented focusing on environmental aspects, with the environment referring to both physical and natural environments. Second, results were also classified along social dimensions (the effect of sport on quality of life and social relations). Third, we highlighted the potentially most important aspect of doing sport and physical activity, which is its effect on the individual and the level of health in society in general.

## 3. Results

### 3.1. Associations between Living Environment and Physical Activity or Sport

The location of our living environment and the layout of the neighborhood are of major importance regarding our willingness to exercise. When sports facilities are eye-catching in a given neighborhood, inhabitants will be more likely to engage in sport simply due to the visual stimulus [33]. Otherwise, people will easily find an excuse to avoid doing so; for example, many complained of traffic issues hindering them when wanting to join sports programs [34]. The availability of sports facilities largely depends on the socioeconomic status of the examined living environment: the lower the status, the lower the availability of sports facilities was; also, the higher status the neighborhood had, the higher the availability of sports facilities, especially privately owned and seasonal sports facilities, was [35]. In districts with a worse social environment, community spaces that can also be used for doing sports are in worse conditions, and they are less clean and less well-maintained compared to richer city districts; consequently, the inhabitants are less satisfied with these services and show a decreasing tendency towards being engaged in some sport [36]. At the same time, it was also observed that children coming from families of lower socioeconomic status play sports less frequently than children living in families with a higher socioeconomic status. In addition, as children that are not participating in any sports in childhood will be even less likely to pursue some sport in their teenage years [37], these children living in neighborhoods of a lower socioeconomic status will rarely become adults that play some sports regularly. Among the people exercising regularly in adulthood, the proportion of those who also kept playing some sports in adolescence is a lot higher, which implies that childhood sport activities which continued in teenage years would likely result in these people doing physical activities in adulthood as well. Therefore, it is particularly regrettable that involvement in physical activities drops drastically in adolescence; also in a significant number of cases, physical activities will be limited almost exclusively to that involved in household chores and active transportation, e.g., walking or cycling to and from school [38]. Several studies confirm that people of a higher socioeconomic status (SES) are more active physically [39], as this favorable status leads to having more opportunities in terms of proper infrastructure and the facilities needed for physical activities. Thus, these people tend to achieve a moderate to intense physical activity level doing 5 or more hours of sport weekly, which, in turn, decreases their prevalence of obesity [40]. Nevertheless, in neighborhoods of a lower socioeconomic status, sport is a means of development. Sport as an activity capable of shaping community identity can serve as grounds for the operation of social institutions that support the sustainable development of individual city districts. Results bring about further results, as communities that have once started a successful initiative will be able to launch further successful projects [41]. For doing physical activities, it is essential to have an appropriate number of and high quality community sport spaces; it is essential that the sports designed to be pursued there fit the expectations of the given community; it is also essential for the place to be reachable within 10 minutes for a large portion of the population [42]. This is extremely important because sport as an activity takes up part of people’s limited free time [43]. Physical activity can develop not only physical but mental health as well, especially in children. The increase of physical activity in youth can lead to higher academic performance at school, improved cognitive functions, and better behavior [44], which may indirectly help children to increase their material prosperity later. Lee and Lim [45] highlighted that sport is also beneficial in sociological terms, especially for the young, since sport teaches them rules and discipline. They used data on 2300 young Koreans to study the correlations between sports activities, the social environment, and juvenile aggression. Their results show that sport activity affects juvenile aggression through a sustainable social environment. Exercising more had no direct effect on juvenile aggression, but could improve teenagers’ relationships with teachers and friends, which in turn could reduce juvenile aggression. 

The data in Table 2 show that, with certain limited exceptions, the number of people engaged in physical activities decreases with age. In countries where the rate of engagement was low at a younger age, the score remained low at older ages as well. The data also demonstrate that the western European population shows a higher tendency to do physical activity compared to Eastern Europeans; meanwhile, northerners are, actually, more active than southerners. The situation is especially alarming in Hungary, Romania, and Turkey, where none of the age groups above 20 reported participation rates in physical activity of more than 10%. Their rates largely depend on the income situation of the region, as well as the prevalent social and cultural views. It is assumed that in places where the desire to increase sport activity is hindered by low incomes, it is extremely difficult to produce changes. However, low level physical activity results in a decline in people’s health and increases in health expenditure. Consequently, conducting health economic studies in the region gains substantial importance [46]. The lack of physical activity results in obesity; childhood obesity in turn leads to an increase in the risk of cardiovascular diseases and diabetes in adulthood, not to mention different long-term socioeconomic consequences [47]. One solution to the problem is to introduce active breaks between school lessons when students can do short but frequent physical exercises to freshen up [48].

Besides serving traffic and transportation aims, city community spaces can also play an active role in the functioning of communities. These spaces provide residents with the opportunity to do special activities like being involved in sport. Such excellent multi-purpose infrastructural developments include bicycle paths [50]. The rate of physical activity is also influenced by the rate of facilities in an area that support active transportation. For example, in areas where more pavements and bicycle paths are built, people will be more willing to walk or cycle. The frequency of use of these infrastructure objects depends on further factors as well, such as what recreational or shopping facilities they provide access to, road safety, the beauty of the scenery, or public safety in the area. Bicycle use also depends on topography conditions, since slopes and hilliness may deter many persons from choosing cycling as a physical activity [51]. Recreational and commuter cycling are influenced by various social and physical environment factors. The social factor that has the biggest impact as a motivational factor on recreational cycling is having friends who also cycle, that is—independently of sport—friends with similar interests, cycling communities (for example clubs), a cycling partner, or family members. Out of the social factors that mostly influence transport cycling, the following ones must be highlighted: social norms on cycling, knowing other cycling commuters, the local transportation culture, and drivers’ attitude. Regarding physical environment factors that influence recreational cycling, the most important ones are weather, topography, cycling safety, route connectivity and accessibility of public transit services, and the beauty of the scenery. Some of these factors, for example weather, topography, or transportation safety, can also be found in the list of factors affecting commuter cycling; this list can be complemented with further factors like bicycle path quality, availability of off-road cycling paths, and end-of-trip facilities for cyclists [52].

When planning modern smart cities and their public utility systems, special attention is given to the fact that the most important public services—including the infrastructure necessary for sports—must be accessible both by means of public transport and bicycle. Furthermore, each settlement should have their own local bicycle use strategy [53]. The architectural structure of educational facilities (schools) also plays an important role in settlement planning due to the impact it has on children’s and adolescents’ willingness to engage in physical activities. The rate of physical activity in children attending schools that provide proper quality sports facilities is definitely higher; also, where students are required to spend time in the open air where they can use part of the infrastructure without being attended to, the rate of physically active children is even higher [54]. It has been observed in several parts of the world that an increasing proportion of the population has been doing physical exercises in multi-purpose community spaces like parks or public squares [55]. Therefore, urban planning should also focus on developing these spaces in such a way so that they would be suitable for physical activities and are easily accessible for the population at the same time. The spatial distribution of sports facilities and the types of activities available in the different institutions and establishments have a major impact on their utilization rate and the subsequent maintenance of the assets. Experience reveals that some sports facilities are overloaded, while others could be visited by a much higher number of persons than they actually are. The utilization rate of sports establishments highly depends on their geographical location, accessibility, and condition, the number of sports that can be pursued there, as well as the extent to which they can meet local requirements [56].

Commitments to sustainability may vary markedly from person to person (and the same is true at the level of organizations, settlements, regions, countries, etc.). Sport environmental citizenship implies caring for the environment, being concerned and taking action to preserve the natural environment and maintain environment sustainability. Basically, any sports company, sports association, counselling, national or local government body, establishment, event or person—including sports people—can be a sport environmental citizen, and support the objective of sustainability in sport [57]. Even though sports facilities, which have certain impact on the environment any way, are operated by skilled staff who will ideally do everything possible to reduce the negative effects these facilities have on the environment, they still cannot be efficient alone [58]. However, the greatest sports events such as the Olympic Games or the Commonwealth Games can promote commitment to sustainability due to their numerous positive social effects including the promotion of volunteering, strengthening the willingness to engage in physical activities, as well as the promotion of well-being and a healthy lifestyle. Such events and phenomena are quite beneficial for raising awareness and turning people’s attention towards sustainability [59]. Furthermore, corporate social responsibility activities, which are gaining more and more popularity, also have the potential to induce positive changes: by organizing different programs and supporting sport initiatives, these organizations can change people’s attitudes towards physical activities, and enhance community cooperation. As a result, such sports events can increase the population’s awareness regarding the environment and sustainability [60]. Moreover, it is also worth mentioning the engagement of famous sports clubs and their actions taken to promote sustainability. Nowadays, an increasing number of professional sports persons, teams, and leagues are devoting particular attention to important societal problems, including the promotion of sustainability. The tools they employ to achieve such aims vary to a great degree; it also happens that individuals involved in different competitive sports cooperate in activities designed to change the image of a settlement to reflect their commitment to environmental sustainability. As a result, residents obtain a more sustainable environment to live in, which supports physical activity as well [61].

In the past years, researchers [62,63,64] have been paying special attention to the study of correlations between physical inactivity and the health damage it causes. The problems arising from this considerable increase in health damage impose a significant public health burden on various policies; therefore, more and more studies are being published on the inherent positive effects that our built and natural environment can exert on us. Some studies focused on the exploration of the correlations between the environment and physical activity and used their results in transportation planning and urban development, by creating recreational parks, bicycle paths, and green areas, and by developing design elements aimed at promoting and supporting active and healthy lifestyles among residents [65,66,67,68,69,70].

The relationship between the environment and human behavior has long been studied by researchers [71]. However, since studies on the correlations between physical activity and built environment have only recently taken off [72,73,74], this field of study is not so thoroughly explored. Yet, several studies focusing on built environment and residents’ physical activity have confirmed that the built environment is a positive determinant of moderate physical activities like walking [39,75,76,77,78,79,80]. Brymer et al. [81], for example, claimed that extreme sports—which are usually considered as risky sports done in a fight against Mother Nature—improved the relationship between individuals and nature. This may mean that individuals become more committed to environment protection, which in turn may result in more sustainable environment-related practices.

### 3.2. Green Spaces as Settings for Physical Activity and Sport

Nowadays, green exercise is gaining more and more popularity, as it can enhance physical activity in all age groups. Most studies explore outdoor exercise along three dimensions. First, research [82,83,84,85] compared the results of outdoor exercise done in a built environment to exercise in a “more nature-like” environment. The second dimension compared the results of indoor and outdoor exercises; out of these, Ryan et al., Focht, and Kerr et al. [86,87,88] must be highlighted. Regarding studies done on effects resulting from changes in the visual environment in laboratory settings, Akers et al. [83] and Pretty et al. [89] must be mentioned. 

However, while the importance of any activity done in the open air is an often-investigated topic, it still must be further emphasized because as a result of global population increases, more people are moving to urban areas. Urbanization is usually described in terms of population density and information on the city size growth rate [90]; this rate has been continuously increasing since the 20th century [91]. The population is expected to continuously grow in the 21st century, with more and more people moving to cities; thus, the study of the population’s spatial distribution is essential for a better understanding of urbanization processes [92]. Interestingly enough, [79] found a positive correlation between residential density and physical activity, whereas several other studies observed no correlation between these factors [93,94,95].

Big cities are also responsible for a considerable part of global greenhouse gas emissions [96]; moreover, cities provide a market with ever increasing demand for goods which are produced in agricultural lands and deforested areas that could otherwise do greenhouse gas sequestration [97]. Therefore, in terms of global sustainability, it is also a priority to stop—or at least mitigate—the flow of rural population into cities. To this aim, it is worth considering what factors could affect young adults deciding whether they should stay in a rural area or move to a big city. Although the notion of the countryside has several negative connotations [98], rural areas still have the potential to retain and attract residents. One of the most frequently mentioned obstacles of rural life is the lack of opportunities, especially the lack of well-paid jobs; on the other hand, an important attraction may be the opportunity to do various physical activities and sports, which also plays an important role in forming community identity [99]. Games, many of which are predecessors of modern sports [100], have always played a major role in rural life, as historical evidence shows. Today, in most rural communities, sport provides the most important grounds for social interaction [101]; thus, the presence of motivating local sport leaders is essential in rural communities’ life [102]. What we can see, however, is that rural regions generally lack competent sporting professionals [103]. Physical activity is a great aim in itself due to its numerous benefits, including for health preservation. Besides, it can support the achievement of some surplus targets [104]. In conclusion, sport makes a major contribution to the prevention of rural depopulation and for achieving sustainability. 

Urban gardens are a further important factor in health development; meeting the needs of urban residents for increasing green space provision and health risks related to urban life are now a research imperative [105]. Therefore, we find it encouraging that physical activity done in green urban spaces has been in focus for a couple of years. Related research [106,107] has found that a number of social benefits regarding psychology, physiology, and general well-being result from such activities. Earlier study results are further supported by Araújo et al. [108], who aimed to explore why participants in outdoor physical activities felt better compared to those who performed activities indoors. Their research found that the remarkable advantage of outdoor activities is that they are affordable and varied. The positive impact of a green environment both in urban and rural areas on mental health was emphasized in Dzhambov et al. [109], Audrey et al. [110], Dean et al. [111], Pretty et al. [112], and Morris [113]; this latter study was complemented by Puett et al. [114] and Thompson et al. [115] saying that the green environment can help individuals to restore their mental strength more easily and quickly, and reduce stress levels in their life; it improves their mood [116], enhances their self-esteem [117], and leads to feeling committed in a positive sense [115].

Research has unanimously demonstrated that exercises done in nature (“green exercises”) provide us with several health benefits. Nevertheless, few studies have addressed the question of why people move outdoors. A Norwegian study [118] explored the significance of nature experiences among motivations for physical activity compared to motivations for sport training. The study found that during physical exercises, nature experiences were considered to be the second most important motivation, which was only preceded by comfort factors. Thus, for persons doing physical activities in green spaces, the most important motivations were comfort and enjoying nature, while sports people considered physical health and socialization to come first.

Due to changes in the social and economic environment in the last two centuries, walking has shifted from being the “central mode of transport” to a “leisure activity”; nowadays it is the most popular physical activity done for pleasure [113]. The popularity of walking and cycling is due to the fact that they are easily available for the majority of the population, and the risk of injuries associated with them is rather low [66]. The studies conducted in the elderly aimed to assess their walking activity, and the accessibility of parks and open-air recreational spaces. Results show that improvements in the quality of neighboring natural green spaces as well as their accessibility enhances open-air physical activity [119,120,121]. Other studies examined the infrastructure supporting walking as well as the environment in relation to residents developing different diseases [40,122]. In this context, developing an urban environment that promotes physical activity may be of cardinal importance in terms of prevention. Cranney et al. [123] draws attention to the potential in outdoor gyms. These open-air gyms increase the active use of city parks, especially if users are reached through target marketing, and receive education on the use of these installations. Eigenschenk et al. [124] also aimed to explore the social benefits that green exercise brings about. They grouped beneficial effects into six categories: physical health, mental health and well-being, education and life-long learning, active citizenship, reduction of crime and antisocial behavior, and other benefits. The results showed that open-air sports help persons to adopt and maintain lifelong physical activity. The reason behind this is that open-air sports are appealing and available for a broad spectrum of people, and investments in outdoor facilities are cost-efficient. 

Plenty of research has been conducted into the relationship between land use mix diversity (meaning how long it would take to get to the shops, the post office, a restaurant, etc.) and physical activity [125,126,127,128,129]. Christian et al. [128] demonstrated (N = 1798) that participants in highly walkable neighborhoods did twice the amount of walking as respondents in neighborhoods with less favorable conditions for walking (low walkable neighborhoods). Santana et al. [130] and van Lenthe et al. [131] found a positive correlation between easy accessibility of shops, service providers, and workplaces, and physical activity.

Looking at statistical data, we can draw the conclusion that the trends depicted above are also supported by the 2018 Eurobarometer data. While the majority of European citizens prefer exercising in the open air, more than half of Hungarians doing physical exercises prefer a home environment, in alignment with the residents of other Eastern European countries. An American study [99] conducted in 2014 with 11,649 participants included (men and women above 20), which aimed to explore the settings for physical activity, proved to yield similar results to the data seen in the Eurobarometer. The data were gained from questionnaires and related clinical assessments. A total of 18% of study participants exercised indoors, 54% outdoors, and 28% in both environments. Participants who exercised partially or entirely outdoors were more frequently physically active. The study also observed that participants that engaged in outdoor activities had better stress management, whereas indoor physical activity environment may be more important for not very active populations. 

### 3.3. Social and Environmental Development Aspects of Physical Activity and Sport

The results of the Eurobarometer carried out in 2018, which are summarized in Table 3, show that the lowest number of persons exercising outdoors can be found in Hungary, whereas 67% of the population of Finland prefers to do exercises in the open air. It is also clear that more exercise is done while going home, to work, or to school. Comparing the data of the last two surveys, we can see that the rate of those exercising in home environment shows a further increase. 

The popularity of gyms has increased by 3%, and 6% more Europeans prefer doing sports at their workplace. This may be due to the fact that an increasing number of multinational companies consider it important to support sports and provide sports facilities [132,133].

The Global Action Plan on Physical Activity launched by WHO in 2019 set out four strategic objectives, which provide a general framework for multidimensional policy making. The goal of this strategy is to increase physical activity and reduce sedentary behavior. The action plan aims to achieve a relative reduction of 15% in the global prevalence of physical inactivity compared to 2016 data among adults and children alike. The four strategic objectives are as follows:

- Create an active society (Create a paradigm shift in all of society by enhancing knowledge and understanding of the multiple benefits of regular physical activity at all ages.)
- Create active environments (Create and maintain good quality public and green, recreational spaces for all people, of all ages and abilities, living in urban or rural communities, for them to have equitable access to safe sport places and facilities.)
- Create active people
- Create active systems.

At the 2015 summit, the United Nations adopted the 2030 Agenda for Sustainable Development, which set 17 sustainable development goals. The United Nations Office on Sport for Development and Peace focuses on the close relationship between sport and sustainability. Sport can serve as a pillar for peace and development, since it plays an essential role in nations and communities showing respect and tolerance towards each other, in improving the situation of communities and individuals, especially women and young people, as well as in enhancing health, education, and social inclusion. All development goals are closely related to physical activity and nutrition targets [134]. Lindsey–Darby [135] considers this to be the first instance when such a broad development policy included sport as well, thus recognizing the role of sports in development. At the same time, authors also highlight the challenges to the achievement of these goals; addressing the complexities within and between countries makes attainment of policy coherence considerably more difficult. 

**Figure 1:**
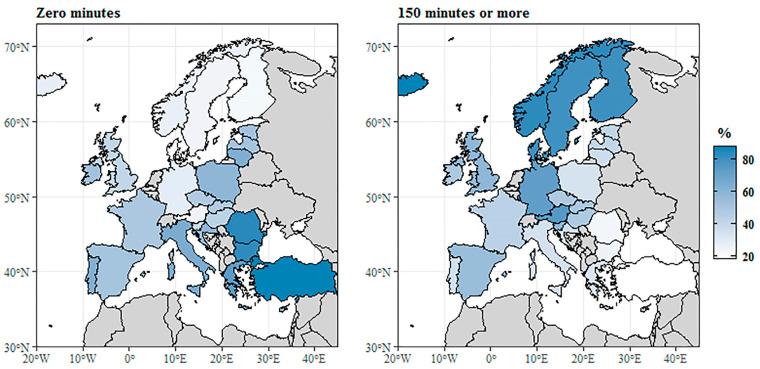
Time spent on health-enhancing (not work-related) aerobic physical activity (2014). Source: [136].

The map in Figure 1 shows the distribution of time spent on health-enhancing activities for the two extremes. The map on the left shows the share of persons who spent no time on these activities, while the map on the right shows the percent of individuals who claimed to be engaged for at least 150 min. The “least active” country was Turkey with nearly 88.2% of its population not setting aside time weekly for health-enhancing physical activity. The share of inactive people was also high in Romania (84.4%), Bulgaria (82.7%), and Greece (74.1%). The mean for the EU-28 was 48.8%, which was exceeded by Italy, Lithuania, Portugal, Cyprus, Poland, Croatia, Ireland, Estonia, Latvia, Spain, and France. The most active country proved to be Denmark, where only 18.7% of the population did not spend time weekly on physical activity. Finland, Sweden, Austria, Norway, Iceland and Germany also had a low share of inactive individuals (23–29%). 

The most active country in this respect was Iceland, where 60.8% of the people spend at least 150 min a week doing physical activities. The value for Norway, Denmark, Finland, Sweden, and Austria also exceeded 50%, which was significantly higher compared to other European countries. The mean for the EU-28 was 30.8%, while the countries at the end of the ranking remained significantly below this value. Turkey had a share of 4.7%, while Romania and Bulgaria had a share of 8–9%. Typically, southern countries like Greece (16.7%), Italy (18.2%), Portugal (18.4%), and Croatia (19.4%) also reported low values; similarly, Poland and Lithuania—although not southern countries—had low percentages of around 17.1% and 19.7%, respectively. The rest of the countries were around the EU-28 mean. In conclusion, considerable differences were observable across European countries with regard to physical activity, which is due to complex economic and sociological factors.

The data presented in the figure illustrate well the presence of an east-west and north-south distribution in physical activity across Europe: southern and eastern EU member state citizens tend to be physically inactive, whereas a larger number of the population of northern and western member states is more likely to spend time exercising. One possible explanation for this is that access to sports may considerably depend on the general economic situation of a society and the social status of its citizens, as early as during childhood [137]. Certain factors, for example one’s social class, may diminish their chances to actively pursue some sport or participate as volunteers in organizing different sports events [138]. Andersen and Bakken [139] draw attention to the difference seen across social groups in terms of participation, with members of higher status social groups participating more in organized sport activities; Gemar [140] adds that commitment to exercise is also influenced by the type of available social capital.

In order to increase sport activity in the population, it would be useful to find an acceptable way to interfere with people’s lifestyles. When discussing sustainability issues related to health, Sansano–Nadalet al. [141] focus on the sustainability of changes in physical activity. According to them, promoting physical activity can lead to health enhancement, which, however, requires maintaining the achieved lifestyle changes. Some people do not need any external motivation for that, as the positive effects of lifestyle change alone provide them with enough motivation to maintain these changes; others need external motivating factors, though.

**Figure 2:**
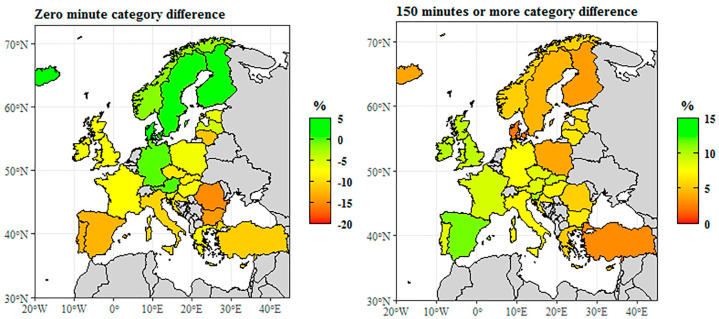
Time spent on health-enhancing (not work-related) aerobic physical activity by sex difference (male **–** female, in %, 2014). Source: [136].

Looking at the prevalence of physical activities across sexes, we found a significant gender gap (Figure 2). Comparing respondents who spent no time exercising, we could see that gender gaps were most pronounced in southern member states. The difference was generally negative in these countries, which means that more men reported spending no daily time exercising. The mean for the EU-28 was −7.5%, which implies that in general, the share of men was higher in the EU. The biggest difference could be observed in Romania (−15.2%), Bulgaria (−14.3%), Portugal (−13.1%), Spain (−12.6%), Turkey (−10.9%), Italy (−10.6%), and Croatia (−9.8%), and a significant difference was also seen in northern countries like Lithuania (−11.6%) and the Czech Republic (−9.1%). In fact, there was no gender gap in Finland, Sweden, Austria, Germany, and Norway, where the difference across sexes was around −1.5% or 1.1%, depending on the country. This means that in the above countries, the share of men spending no time exercising was the same as the share of women not doing any physical activity. A significant positive difference could only be seen in Denmark and Iceland (+4.8%, and +3.2%, respectively), which meant a higher share of men spending no time exercising than women.

The category of at least 150 min includes respondents who spend 150 min or more a week on health-enhancing physical exercises. In this category, no negative difference was observable, which implies that there was no European country with a higher share of women. In Denmark, Turkey, Finland, Iceland, and Poland, the gap remained in general between +2.2 and 3.9%. Some countries like Portugal, the Czech Republic, or Austria reported a difference over 8%, while France, Slovakia, the United Kingdom, and Luxembourg presented a 9% gap. The biggest difference was seen in Spain (+11.4%), Slovenia (+10.6%), and Ireland (+10.1%), with men’s higher share. This was quite close to the EU-28 mean, which was 8.1% in 2014. These data demonstrate altogether that women in general were more likely not to take part in any health-enhancing, not work-related physical activity. This was especially true for southern EU countries, and more to the north we looked, the more this tendency declined; in the northern EU, this trend was reversed. In the category of at least 150 min a week, there was no such obvious geographical difference, and the data distribution was not so significant either—this means there was no significant gender gap. Nevertheless, wherever there was a gap, it was men who had a higher share.

**Figure 3:**
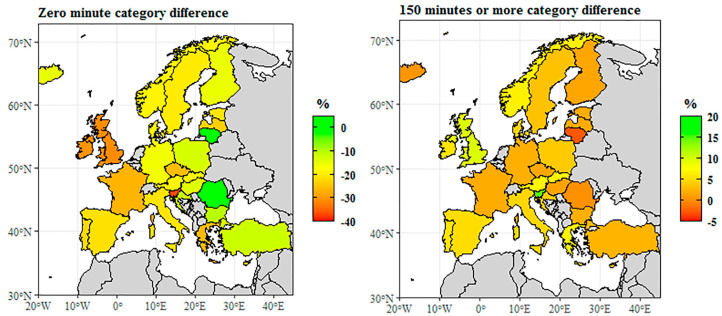
Time spent on health—enhancing (not work—related) aerobic physical activity by educational attainment level difference (male—female, in %, 2014). Note: Educational attainment level according to the International Standard Classification of Education (ISCED 2011), where levels between 0 and 2 means less than primary, primary, and lower secondary education and levels between 5 and 8 means tertiary education. Source: [136].

The level of educational attainment also influences the level of participation in physical activity or the attitudes towards it. Differences were analyzed across two groups. The first group included the population who did no health-enhancing physical activity during a week, while the other group consisted of individuals who exercise at least 150 min a week. We analyzed in both groups whether there were any differences between those with 5–8 level (tertiary) and those with 0–2 level (less than primary, primary, and lower secondary) education (Figure 3).

Significant differences could be detected between the levels. In Slovenia, the United Kingdom, and Ireland, the difference between the shares peaked at −30% percentage points, that is the share of those with 0–2 level education was much higher in these countries. The difference between the shares of the two educational attainment levels also exceeded −20% percentage points in several countries, namely Luxembourg (−29.8%), France (−26.9%), Greece (−26.2%), and the Czech Republic (−25.8%), where there was a difference of −25% percentage points; and Latvia, Portugal, Spain, Italy, Estonia, and Sweden also reported a difference of over −20%. The mean of the EU-28 was −27.8%, which also proved to be significant compared to the indicators of the individual countries. Only two EU countries reported positive differences. In fact, no difference was seen in Romania (0.2%), and the difference in Lithuania was also as small as 2.1%. This means that among those spending no time per week on physical activity, the proportion of low educated people was generally higher. Among those spending at least 150 min per week performing physical exercises, there was no such significant difference. Only Lithuania reported a negative difference, with the proportion of those with 0–2 level education being slightly higher. Several countries recorded practically no difference. The difference in Romania, Iceland, Hungary, Finland, and the Czech Republic was below +1.5% percentage points, and the difference in Latvia, Estonia, France, Germany, Bulgaria, Luxembourg, and Turkey also failed to exceed +3%. The highest positive difference could be observed in Slovenia (+15.3%), and it was also significant in the United Kingdom (+9.8%) and Malta (+7.8%). The EU-28 mean difference was 7.2%, implying that among those spending more time on physical exercises, the proportion of individuals with tertiary educational attainment was slightly higher. No obvious geographical tendency could be observed in any of the cases.

As a general remark, it can be said that men tend to spend more time weekly on health-enhancing physical activities. The rate of women spending no time on physical activities is significantly higher, which is especially true in southern Europe. In addition, persons with less than primary, primary, or secondary educational attainment have a higher share among those who spend no time exercising. This can result in remarkable differences across countries. However, in case of the category with at least 150 min of exercise, there were not very significant differences across member states. Besides the above, no significant differences across categories could be seen in terms of gender or educational attainment. The question why the level of physical activity was lower in women and persons with primary or secondary educational attainment is difficult to answer. Presumably, women’s results may have been influenced by their situation of having children and the resulting lifestyle. Regarding educational attainment, the amount of time spent exercising may have been affected by respondents’ job type, or material considerations.

In our article, we used several factors related to the environment, sports, and sustainability (Table 4). As a part of the discussion, we also paid attention to the possibilities available to change them. We examined the socioeconomic status as a factor influencing the participation in physical activities and the attitude towards sustainability and we found that there is a close relation between sports and sustainability. The socioeconomic status could be changed on individual, regional/national, and global levels as well. We also found that there are several factors which are changeable on the regional/national level like the visual surroundings, the quality and the quantity of the places where people can do sports, and the summarizing category of urban infrastructure including common spaces, sidewalks, and bicycle paths. There are also some factors which people can change on individual, on regional/national, and on global levels. We listed these factors as the following: sport environmental citizenship, awareness regarding the environment and sustainability, green spaces, and outdoor activity. In case of some other factors like the indoor and outdoor exercises or like the land use diversity, people only have the chance to change them on individual and/or on regional/national levels.

## 4. Discussion

The analysis into relationships between sport and sustainability highlighted the importance and effect of the physical living environment, natural environment and socioeconomic environment. Based on our results, the economic status seems to be the most determining factor at both the social and individual levels. The study of the social environment shows that the residents of higher social as well as economic status places [35,37] are more willing to be engaged in sports. The populations of southern and eastern EU member states engage in lower levels of physical activity than citizens of northern and western member states, the latter of which have a better economic status. At the individual level the importance of the economic situation is also evidenced, because people with higher education are likely characterized with a better employment and financial situation, and are found to be more likely active than those with a lower educational attainment.

Based on our results, better access to the sports facilities is related to more physical activity. This should direct the attention of decision makers towards the availability of infrastructure, not only involving the availability of sports facilities but also the availability of multi-purpose community places, or transportation facilities.

When sports facilities are eye-catching in a given neighborhood, residents will be more willing to play some sport simply due to the visual stimulus [33]. An increasing proportion of residents do exercises in multi-purpose community places, parks, or public squares [55]. Residents’ physical activity is also influenced by the availability or lack of transportation infrastructure and facilities [39,75,76,77,78,79,80,142,143]. In areas where more pavements and cycle tracks are built, people will be more willing to walk or cycle [50]. By now walking has shifted from being the “central mode of transport” to “leisure activity” [113]. In areas of high walkability, walking levels doubled [128]. When planning smart cities, special attention should be given to making sure that the infrastructure necessary for sports is available and accessible both by means of public transport and by bike [53]. 

Urban population is also characterized by an increasing demand for green spaces and natural environment [105] Besides the health benefits associated with open-air exercise [82,83,84,85,86,87,88], research also found social benefits closely related with one’s psychological [109,110,111,112,113,114,115,116,117], physiological, and general well-being [106,107,108]. In terms of motivation to exercise in natural environments, nature experiences were usually the second most important motivation, which was only preceded by comfort motives [118]. Another advantage of outdoor physical activities is that they are affordable and diverse. Open-air sports help persons to establish and maintain lifelong physical activity; these sports are appealing and available for a broad spectrum of people, thus investments in outdoor facilities are cost-efficient.

## 5. Conclusions

Our research has confirmed the correlation between sport and sustainability by exploring the relevant literature background. As a conclusion, we can state that the built environment is of paramount importance in establishing and maintaining physical activity. Apart from establishments providing sports opportunities, multi-purpose community spaces and transportation infrastructure can also promote physical activity. Natural environment has also proved to be a significant motivation for movement activities. In social terms—be it in a community or a household—higher status is a predictor of higher physical activity.

Programs aimed at improving physical activity are markedly important for those living in eastern and southern Europe, also for women (regarding gender), and for persons with a lower educational attainment.

The present study demonstrates that increasing physical activity will subsequently lead to both a direct and indirect increase in the awareness of the population towards environment and sustainability.

In our research, we find that in addition to the global development plans of WHO and the United Nations, most intervention options are possible at the regional/national level. The local/national governments need to create sports facilities, which are easily available for the majority of the population. Our results show that urban, common, multi-functional and green spaces, sidewalks, and bicycle paths can be significant in terms of physical activity. From this aspect, developing an urban environment that promotes physical activity may be of cardinal importance in terms of illness prevention measures.

In the era of the novel coronavirus (COVID-19), we can see the importance of possibilities related to outdoor activities. As more and more lockdowns were prescribed globally, more and more people realized how limited our possibilities are regarding nature and the environment. This phenomenon also raised awareness on the issues of sustainability in connection not only with environmental aspects but also on its effect on personal wellbeing as well. When the strict regulations made it possible, people tried to do physical activities in nature and this tendency can have a long-term effect on the ways that people complete physical activities.

## Figures and Tables

**Table 1:** Time use in percentages among the 15–64 age group (%).

Country	Paid Work or Study	Unpaid Work	Personal Care	Leisure	Other
Australia	16.54	16.89	45.66	19.53	1.39
Austria	21.28	14.03	43.89	20.22	0.58
Belgium	16.37	13.32	46.11	23.57	0.64
Canada	21.16	12.92	44.21	19.37	2.36
Denmark	15.61	15.04	45.79	22.84	0.72
Estonia	17.75	14.42	46.02	21.49	0.35
Finland	15.91	13.67	44.48	22.99	2.95
France	14.18	12.58	52.24	20.38	0.62
Germany	17.21	13.61	45.00	23.00	1.18
Greece	15.81	12.51	47.54	23.71	0.42
Hungary	16.50	15.91	47.44	19.54	0.61
Ireland	18.61	14.56	42.29	21.69	2.86
Italy	12.27	15.21	49.15	22.45	0.92
Japan	25.18	9.17	43.06	19.33	3.27
Korea	23.92	9.15	47.07	17.93	1.93
Latvia	22.91	13.62	44.53	18.67	0.27
Lithuania	21.89	15.59	44.83	17.13	0.57
Luxembourg	19.77	12.44	47.62	19.19	0.98
Mexico	24.25	18.33	44.00	11.94	1.49
Netherlands	16.86	12.89	47.33	21.86	1.06
New Zealand	18.75	14.17	45.00	20.90	1.18
Norway	16.75	13.61	43.56	25.57	0.50
Poland	18.03	15.69	45.63	19.87	0.78
Portugal	20.49	15.45	46.92	16.71	0.42
Slovenia	18.38	16.02	43.62	21.57	0.42
Spain	14.03	15.05	48.04	21.95	0.93
Sweden	20.38	13.65	43.23	22.33	0.43
Turkey	17.11	12.92	47.35	19.84	2.78
United Kingdom	18.21	13.51	44.81	21.22	2.25
United States	19.62	13.45	45.01	20.24	1.67
China	23.59	11.38	48.16	15.82	1.04
India	20.48	13.27	47.74	17.64	0.86
South Africa	16.71	12.66	48.27	21.24	1.12

Source: [32].

**Table 2:** Participation rate in sports and outdoor activities (except walking and hiking) by age group (%).

	From 15 to 20 Years	From 20 to 24 Years	From 25 to 44 Years	From 45 to 64 Years	65 Years or Over	Total
Belgium	18.1	14.3	12.4	10.5	9.9	12.0
Germany	21.8	21.3	16.1	17.9	23.9	19.3
Estonia	25.1	19.6	14.8	13.6	19.6	16.7
Greece	24.7	21.6	13.2	5.6	2.7	9.7
Spain	25.2	17.5	13.0	10.1	6.9	12.0
France	17.6	15.2	11.3	9.3	8.1	10.7
Italy	20.6	16.0	10.1	7.8	6.3	9.6
Luxembourg	24.5	25.0	18.8	15.5	13.3	17.9
Hungary	16.8	9.5	7.5	4.7	3.0	6.6
Netherlands	21.4	19.9	14.7	15.0	18.6	16.5
Austria	18.1	14.8	14.7	17.3	19.0	16.7
Poland	20.8	15.1	10.3	8.5	8.7	10.6
Romania	13.8	7.5	2.6	1.1	0.8	3.2
Finland	27.6	24.1	21.1	24.0	28.7	24.6
United Kingdom	21.2	20.0	16.2	11.0	10.5	14.1
Norway	29.1	18.7	18.3	19.0	16.5	19.6
Serbia	22.3	18.1	8.8	3.7	2.0	6.8
Turkey	8.0	5.4	3.1	2.4	1.4	3.7

Source: [49].

**Table 3:** Sports venue preferences in EU-28 and Hungary (%).

	EU-28 (2014)	EU-28 (2018)	Hungary (2014)	Hungary (2018)
outdoors	40%	40%	16%	17%
home	36%	32%	51%	59%
transport	25%	23%	29%	29%
fitness center	15%	15%	6%	9%
workplace	13%	13%	13%	19%
sports club	13%	13%	5%	5%
sports center	8%	12%	3%	4%
university, school	5%	5%	5%	4%
spontaneous	4%	5%	3%	3%

Source: [131,132].

**Table 4:** Examined factors in terms of environmental-sports-sustainability and the possibility of changing them on individual, regional/national, and global levels.

Examined Factors	Sources	Level of Intervention		
		Individual	Regional/National	Global
Socioeconomic status	[35,36,37,39,40,41]	+	+	+
Sex	[132,133]	−	−	−
Age	[44,45]	−	−	−
Education	[132,133]	+	+	−
Income status	[132,133]	+	+	+
Visual surroundings	[33,83,89]	−	+	−
Quantity and quality of social sport places	[36,42,56,75,76,77,78,79,80]	−	+	−
Urban infrastructure, common spaces, sidewalks, bicycle paths	[50,51,52,53,54,55,65,66,67,68,69,70,142,143]	−	+	−
Sport environmental citizenship. Awareness regarding the environment and sustainability	[57,58,59,60,61,81]	+	+	+
Green spaces, outdoor activity	[82,83,84,85,106,107,109,110,111,112,113,114,115,116,117,118,119,120,121,122,123,124]	+	+	+
Indoor and outdoor exercises	[86,87,88]	+	+	−
Land use mix diversity	[125,126,127,128,129,130,131]	+	+	−

Note: + = intervention is possible; − = intervention is not possible.

## Data Availability

Not applicable.
